# Non-Invasive Monitoring of Human Health by Photoacoustic Spectroscopy

**DOI:** 10.3390/s22031155

**Published:** 2022-02-03

**Authors:** Yongyong Jin, Yonggang Yin, Chiye Li, Hongying Liu, Junhui Shi

**Affiliations:** 1 College of Automation, Hangzhou Dianzi University, Hangzhou 310018, Zhejiang, China; yyjin@hdu.edu.cn; 2 Zhejiang Lab, Hangzhou 311121, Zhejiang, China; yinyonggang@zhejianglab.com (Y.Y.); chiye.li@zhejianglab.com (C.L.)

**Keywords:** photoacoustic spectroscopy, non-invasive monitoring, glucose, atherosclerosis, blood oxygen, tumor

## Abstract

For certain diseases, the continuous long-term monitoring of the physiological condition is crucial. Therefore, non-invasive monitoring methods have attracted widespread attention in health care. This review aims to discuss the non-invasive monitoring technologies for human health based on photoacoustic spectroscopy. First, the theoretical basis of photoacoustic spectroscopy and related devices are reported. Furthermore, this article introduces the monitoring methods for blood glucose, blood oxygen, lipid, and tumors, including differential continuous-wave photoacoustic spectroscopy, microscopic photoacoustic spectroscopy, mid-infrared photoacoustic detection, wavelength-modulated differential photoacoustic spectroscopy, and others. Finally, we present the limitations and prospects of photoacoustic spectroscopy.

## 1. Introduction

Many people are suffering from various diseases worldwide, including diabetes, cardiovascular diseases, cancer, etc., which cause death to tens of millions of people every year [1,2,3]. These diseases can be prevented or managed through regular monitoring, which improves patients’ prognoses. For example, diabetes is closely related to blood glucose, as it is manifested as a long-term blood glucose level disorder [4]. Therefore, the long-term monitoring of blood glucose levels is critical for the treatment of diabetes. Although a method based on enzyme and electric measurement was developed for monitoring blood glucose, it requires finger pricking [5] and applying blood drops on disposable test strips, which brings about pain and the risk of infection [6]. In addition, since a test strip is required for each measurement, it increases the cost and leads to economic burden for the patient [7]. For cardiovascular disease, the common detection methods are X-ray fluoroscopy and angiography. However, the visibility of these technologies is usually poor [8], which is inconvenient for diagnosis or treatment. X-ray angiography techniques use iodine-based contrast agents to acquire vascular angiograms, meaning that iodine-based contrast media must be injected into the blood vessels [9]. The method involves puncturing the blood vessel, increasing the risk of bleeding and infection [10]. The treatment of cancer at an early stage is an effective strategy to reduce mortality. By monitoring morphology and the surrounding blood oxygen concentration, cancer can be screened. There are some common medical imaging technologies, such as X-ray imaging, ultrasonic imaging, and MRI [11]. However, the imaging contrasts of certain tumors are not obvious, thus X-ray imaging and ultrasound imaging are not very sensitive. Due to limitations such as low specificity and a high false-positive rate, MRI is also not ideal [12]. In addition, the detection cost of these medical imaging technologies is also expensive. Each CT and MRI examination costs hundreds of dollars [13,14], which brings a great economic burden to patients and the healthcare system. For blood oxygen monitoring, the currently available method is a pulse oximeter. However, its accuracy highly depends on operators’ skills, which affects its reliability [15].

Therefore, people have proposed many methods and technologies over the past decades in order to monitor blood glucose, blood oxygen, lipid, and tumors non-invasively and effectively. These methods can be divided into optical methods and non-optical methods. Optical methods include Raman spectroscopy [16,17,18], photoacoustic (optoacoustic) spectroscopy (PAS) [19,20,21], near-infrared absorption spectroscopy [22,23], and photoacoustic imaging [24,25,26,27,28,29,30]. Non-optical methods are ultrasound [31,32,33] and microwave sensing [4]. The traditional optical technology has the problems of strong light scattering and attenuation in human tissues, which makes the penetration depth shallow. On the contrary, techniques based on PAS are helpful for alleviating this problem, because PAS has the advantages of high optical contrast and deep acoustic penetration [21]. PAS can realize the non-invasive monitoring of human health and minimize the disturbance or harm to patients, while maintaining high sensitivity and specificity. Moreover, it has the potential to be used as a consumer product and thus, would have good accessibility and a low economic burden. Therefore, PAS is becoming more and more popular, and many health monitoring methods based on PAS have been reported.

In the past ten years, extensive research has been carried out to develop a novel PAS diagnostic system for biomedical applications, and there are plenty of reviews that aim to review PAS techniques for health monitoring. For example, Bayer et al. reviewed the application of PAS in cancer detection [34]. Song et al. reviewed the application of photoacoustic imaging technology in microvascular imaging [35]. Erfanzadeh et al. reviewed low-cost photoacoustic tools [36]. These studies show the great potential of photoacoustic spectroscopy in medical areas. However, previous literature reviews mainly focus on a single disease [37,38], and there is no review that systematically introduces the research of PAS in the monitoring methods of major human diseases and their relations with each other. This review aims at familiarizing researchers with the current technical advancements of PAS in human monitoring and discusses future research directions that can promote the development of methods with higher sensitivity and less error, and which also reduce the discomfort and pain of patients. Based on recent research, this paper lists various human health monitoring methods using PAS, including differential continuous-wave photoacoustic spectroscopy, microscopic photoacoustic spectroscopy, mid-infrared photoacoustic detection, wavelength-modulated differential photoacoustic spectroscopy, and other methods. Section 2 introduces the theoretical basis of the PAS technique and related devices. Section 3 shows the research of PAS in blood glucose, blood oxygen, lipid, and cancer cell monitoring, including the research methods, experimental setup, and results. Finally, in Section 4, the advantages, disadvantages, and development prospects of non-invasive monitoring via PAS are discussed.

## 2. Photoacoustic Spectroscopy

The theory of the photoacoustic (PA) effect was discovered by Bell in 1880. When light beam irradiates the tissue, photons can penetrate to a certain depth inside the tissue. They scatter and are absorbed by specific light absorbing molecules known as chromophores. The absorbed energy is converted into heat by vibrational and collisional relaxation. This produces an initial pressure increase and the subsequent emission of acoustic waves. This pressure wave, named the PA signal, propagates to the surface, where they are detected [39]. In 1938, Viengerov first published a paper about using PAS to detect gas concentration [40], which made researchers aware of the application prospect of PAS in material characterization. However, due to the limitations of light sources, PAS developed slowly and did not get a breakthrough in the following decades. In the 1970s, due to the invention of lasers and the development of acoustic sensor technology, PAS flourished. In particular, after Kreuzer performed an experiment that uses a laser in PA gas detection [41], PAS has regained the attention of researchers, making it one of the most promising technologies in the field of biology and medicine.

**Figure 1:**
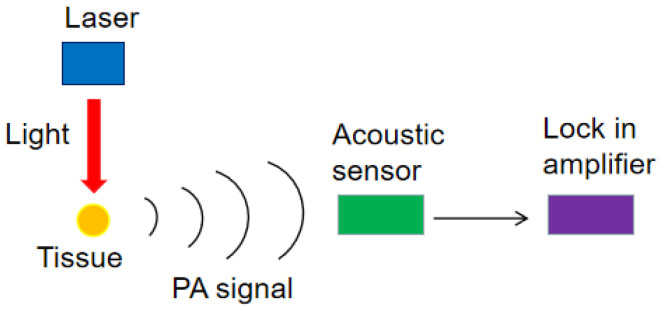
The generation and detection process of the PA signal.

PAS technology uses acoustic methods to measure the light spectrum of samples. Compared with the traditional spectrum detection technology, PAS overcomes the problems of light scattering, reflection, and background interference. As a research tool, PAS has been widely applied in the detection of solid, gas, and liquid in biology, physics, materials science, medicine, etc. A typical system for PAS is composed of lasers, PA cell, acoustic detectors, amplifiers, and a data acquisition unit. The detection process of the target object is as follows: (1) firstly, the target in the PA cell is irradiated with the light of a certain wavelength. After absorbing photons, the target transitions from the ground state to the excited state and then releases heat energy outward. When the incident light is periodically modulated, the temperature of the target will fluctuate accordingly, resulting in a periodic acoustic signal. (2) Then, the acoustic signal in the PA cell is detected by a microphone. (3) Finally, the target absorption information can be extracted by processing the signal. Figure 1 shows the generation and detection process of the PA signal.

## 3. Non-Invasive Monitoring Methods Based on Photoacoustic Spectroscopy

### 3.1. Non-Invasive Monitoring of Blood Glucose by Photoacoustic Spectroscopy

The rapid growth of diabetic patients has become a global problem. When the blood glucose level of the diabetic patient is not controlled, there may be complications such as stroke and heart disease [14]. Therefore, people with diabetes need a regular monitoring tool for their blood glucose levels. At present, the most widely used method of human blood glucose detection is the enzyme test strip method. However, this method cannot measure continuously, and it requires pricking human fingers, bringing physical and psychological harm to patients, and reducing their quality of life. Because of its non-invasive advantage, blood glucose monitoring based on PAS has become a research hotspot.

**Figure 2:**
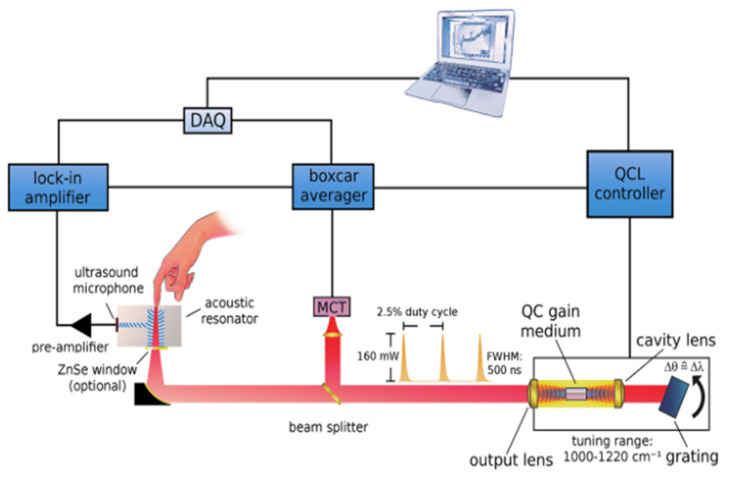
Optical setup and signal detection schematic for the photoacoustic measurement of glucose in skin. Reprinted with permission from [42]. Copyright 2013 American Chemical Society.

Due to the advantages of wide wavelength coverage, high output power, and tunable excitation wavelength, the quantum cascade laser (QCL) has been implemented in blood glucose detection (see Table 1). Pleitez et al. reported the non-invasive determination of blood glucose in the interstitial layer of the human skin by mid-infrared light [42]. They used a tunable external cavity QCL (1000–1220 cm^−1^) to excite the PA signal by focusing on the finger skin. Through the PA spectrum of the detected skin, they achieved a non-invasive detection of blood glucose levels. Although the obtained infrared PA spectrum of the skin is complex, it can be separated into the first principal component that corresponds to glucose. Figure 2 shows the developed optical setup and the signal detection system. The in vivo measurements were carried out on healthy and diabetic volunteers. Comparing the invasive enzymatic test strips method with the non-invasive method, the curve of glucose concentration over time is roughly the same, demonstrating the feasibility of in vivo non-invasive glucose concentration monitoring. However, due to the different skin properties of patients, the measurement results may be biased. Therefore, individual calibration for each patient is necessary for this method. Kottmann et al. implemented a mid-infrared PA setup to track glucose in vitro, using an external-cavity QCL (1010–1095 cm^−1^) and a PA cell with a volume of only 78 mm^3^ [43]. During the experiment, they exposed the epidermis to glucose solutions of different concentrations and continuously ventilated the PA chamber with N_2_ to keep the humidity stable, which ensures that the obtained photoacoustic signal is not affected by water vapor. Their experiments confirmed that there is a short delay between the in vivo blood glucose concentration and the interstitial fluid glucose concentration, and the detection limit of glucose in the obtained skin sample is 100 mg/dL. Furthermore, based on the original research, they transmitted the light of an external cavity QCL (1010–1095 cm^−1^) through a silver halide fiber connected to a PA cell [44]. Through the sensing experiment of glucose in an aqueous solution, the performance of the optical fiber-coupled PA sensor is verified. This study raised the detection limit of blood glucose concentration to 57 mg/dL. Liakat et al. developed a non-invasive in vivo glucose sensor. They utilized a hollow fiber for light transmission, and a tunable external cavity QCL (8–10 μm) to collect the PA spectra of human subjects [45]. By using partial least squares regression, they achieved clinically accurate predictions. Throughout a glucose concentration range of 80–160 mg/dL, they achieved clinically accurate predictions 84% of the time, on average.

**Figure 3:**
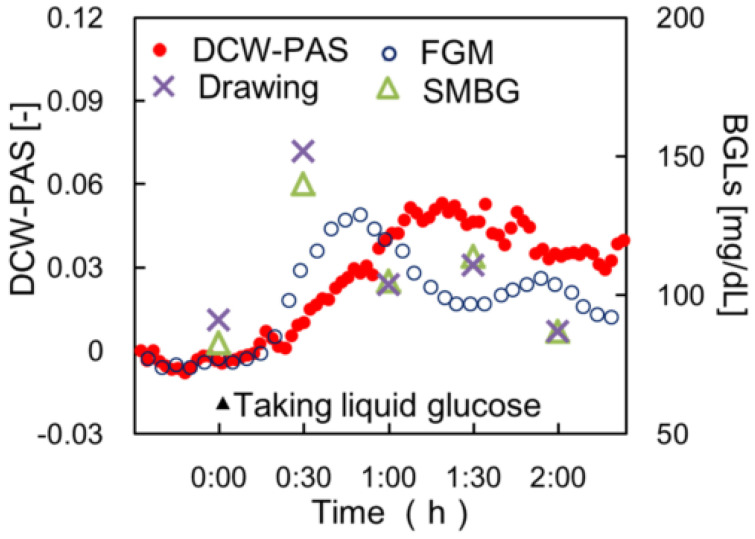
Typical comparison between DCW-PAS signals and the blood glucose levels of volunteers. The closed circles, open circles, triangles, and crosses in the figure are the results from the DCW-PAS, FGM, SMBG, and drawn blood, respectively. Reprinted from [21].

A dual-wavelength laser is also an effective method to detect blood glucose. Tanaka et al. used differential continuous-wave photoacoustic spectroscopy (DCW-PAS) to monitor blood glucose [21]. In the DCW-PAS method, the sample is irradiated by two lasers that are amplitude-modulated at the same frequency but 180 °apart. DCW-PAS is a differential method based on two wavelengths with almost the same absorbance of water but different absorbances of glucose. By adjusting the light intensity of the laser, the glucose concentration can be extracted. In vivo experiment results obtained using this method were compared with a fast blood glucose monitoring tool (FGM), self-monitoring blood glucose tool (SMBG), and the venous blood draw method, which are all invasive. The results are shown in Figure 3. The time sequence of volunteers’ blood glucose concentration detected by DCW-PAS is consistent with the results of the other three invasive blood glucose measurement tools, which shows the potential of DCW-PAS in detecting blood glucose concentration. Utilizing two high-power LED sources emitting at central wavelengths of 444 and 628 nm, Orfanakis et al. developed a compact, economical, and multi-wavelength PA sensing system [46]. An upgraded version of this system may be used to measure various important biomarkers, such as glucose levels, melanin concentration, and oxyhemoglobin saturation, which shows the great potential of a portable PA system.

Methods using a single wavelength to detect blood glucose have also been reported. Zhang et al. reported a novel non-invasive monitoring technique by combining peak-to-peak value and peak arrival time delay from a PA signal. This technique improved the accuracy of blood glucose level prediction without complex instrumentation [47]. When the signal-to-noise ratio (SNR) is high, the technique can achieve excellent prediction accuracy, whereas if the SNR is poor, the efficacy of this technique will be limited. In the latest research, Zhang et al. reported a novel method named time-domain photoacoustic waveform spectroscopy (tPAWS) [48]. This method employs three signal processing tools, MLR, PCR, and PLSR, to extract the information features of multiple variables inherent in the PA waveform excited by a single wavelength laser. Compared to amplitude-based and data fusion methods, this method has higher sensitivity and less error. Moreover, this PA system only requires single wavelength excitation without PA cells or lock-in amplifiers, which is more practical for miniaturization. Zhang et al. developed a miniaturized PA sensor for glucose by integrating the laser source, photo chopper, PA cell, microphone, and laptop [49]. Their PA device is really small in bulk with competitive sensitivity and excellent stability, offering a promising tool for point-of-care testing in bioassays.

Although PAS can be effectively used for the non-invasive monitoring of blood glucose, it is vulnerable to the state of skin, because skin secretions will change the light absorption. Sim et al. combined a MIR-PA sensor with raster scans to investigate the microscopic structure of skin [19]. Position scanning was used to investigate how the signal varied depending on the different positions of the skin. By scanning the secreting and non-secreting regions, the results show that the signal of the secretion strongly interferes with the glucose signal, and the signal of the non-secretion is closer to the reference glucose value from invasive detection. This research provides a reference for subsequent researchers to choose skin areas that are not affected by skin conditions through infrared spectroscopy, in order to reliably predict blood glucose levels. Moreover, the researchers also explored the effect of temperature on the detection in glucose solution. Through experiments, Prakash et al. found that the photoacoustic signal of pure water in the wavelength range from 900 to 1840 nm became mute at 4 ℃, based on which they proposed cooled IR photoacoustic spectroscopy (CIROAS) to mute the contributions of water to the PAS of glucose solution [50]. Compared with traditional PAS, the technique effectively improves the detection sensitivity in glucose solution. Through further experiments, they confirmed that CIROAS can improve protein and lipid detection sensitivity.

### 3.2. Non-Invasive Monitoring of Lipid by Photoacoustic Spectroscopy

Countless people die of cardiovascular disease every year all over the world. Due to its complex clinical characteristics, cardiovascular disease is still a challenge for doctors and researchers. The rupture of vulnerable atherosclerotic plaque is recognized as the major cause of acute cardiovascular events and sudden cardiac death. Vulnerable plaques are characterized by a thin fibrous cap that covers a lipid-rich necrotic core. When the thin cap ruptures, the lipid-rich components in plaques flow into the bloodstream, which leads to the blockage of the rupture site or downstream, resulting in acute cardiovascular disease [52]. Therefore, the lipid of atherosclerosis can be a biomarker to monitor atherosclerosis. In previous studies, researchers used intravascular ultrasound to monitor lipids in atherosclerotic plaques. Intravascular ultrasound generates cross-sectional images of the arterial wall through the reflection amplitude of the ultrasound pulse. However, this technique has limitations in accurately evaluating biological components. Intravascular photoacoustic imaging is a new technique that utilizes the difference of photoacoustic spectra to identify biological components, which can be used to effectively monitor vulnerable plaque. Due to its accuracy and safety, intravascular photoacoustics (IVPA) has attracted more and more researchers.

**Figure 4:**
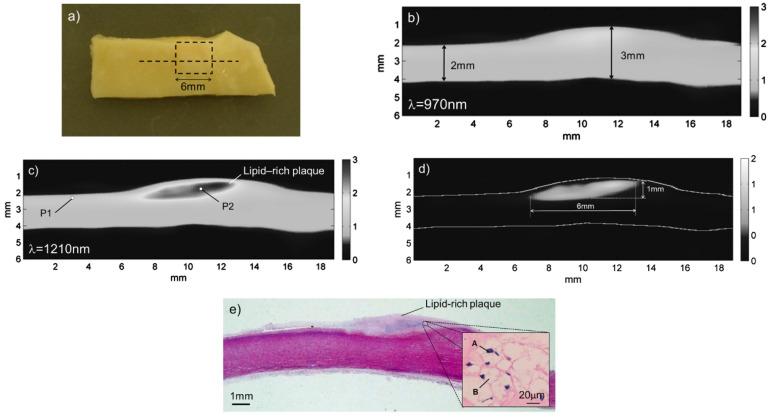
Imaging and sectioning experiments on human aortic samples: (**a**) photograph of a human aorta sample with a raised atherosclerotic lesion located within the dashed rectangle; (**b**) photoacoustic image obtained at 970 nm; (**c**) photoacoustic image obtained at 1210 nm; (**d**) image obtained by subtracting the two photoacoustic images in (**b**,**c**) to reveal the plaque region and overlaid with a segmented image of (**c**); (**e**) photograph of the histological section of the sample. Reprinted from [53].

Here, we summarize the previous reports on the photoacoustic imaging of lipid plaques using lasers at different wavelengths. Allen et al. used the difference in light absorption between lipids and normal arterial tissues in the 740 to 1400 nm wavelength range to distinguish normal tissues from lipid-rich atherosclerotic regions [53]. Using human aortic slices as samples, they measured the photoacoustic spectra of the samples using an acoustic resolution photoacoustic microscope. They imaged the samples at 970 nm and 1210 nm, where the lipids’ absorption of light varies greatly. Then, the differential method is used to subtract the two images, finally obtaining a clear lipid plaque image. Figure 4a is a photograph of the sample. Figure 4b,c shows photoacoustic images of the tissue sample obtained at 970 and 1210 nm, respectively. To remove the contribution of the normal tissue and isolate the lipid-rich region, the images obtained at 970 and 1210 nm were subtracted from each other, as shown in Figure 4d. Figure 4e is a photograph of the histological section of the sample, in which the shape and size of the fat-rich plaque are similar to that in Figure 4d, confirming the feasibility of this technique. Jansen et al. reported a combination of intravascular ultrasound and photoacoustic imaging systems to image human coronary arteries ex vivo [54]. They utilized the relative difference between PA signals at 1205 and 1235 nm for photoacoustic imaging, then superimposed the photoacoustic image on the IVUS image. This technology successfully detected atherosclerotic plaques and peri-adventitia lipids in human coronary arteries. Their research proved that the composition of lipid-rich plaques could be distinguished in cholesterol and a range of cholesterol esters by PA spectroscopy, which opens a new direction for the study of lipid-rich plaques in vivo [55]. In addition, Wu et al. exploited the signal intensity difference of lipids at 1718 and 1734 nm, successfully detecting plaque lipids and peri-adventitial fat in human coronary arteries ex vivo [52]. One study showed that both high absorption bands in the lipid absorption spectrum at 1200 and 1700 nm can be used to distinguish between plaque lipids and peri-adventitial lipids, and the imaging depth of 1200 nm is twice that of 1700 nm [56]. Their techniques can rapidly identify lipid-rich atherosclerotic plaques in human arteries.

Wang et al. reported the selective imaging of biological tissue using a laser with bands of 1600 to 1850 nm [57]. They imaged the arteries of pigs with 1210 and 1730 nm lasers, respectively. It was found that the vibrational photoacoustic (VPA) signal at 1730 nm was six times larger than that at 1210 nm when the blood layer was 0.5 mm thick. Then they utilized 1600 to 1850 nm lasers in the VPA imaging of atherosclerotic arteries through a 0.5-mm-thick layer of whole blood. Iskander-Rizk et al. developed a photoacoustic microscope to extract the photoacoustic spectral characteristics of plaque lipids in human endarterectomy samples in the range of 1150–1240 nm [58]. Four spectral peaks at wavelengths of 1164, 1188, 1196, and 1210 nm were obtained by PCA decomposition. Compared with the results of matrix-assisted laser desorption ionization mass spectrometry imaging, they found that the PA signal of plaque had the best correlation with sphingomyelin and cholesterol ester, which revealed that PAS can be used to detect advanced atherosclerotic plaque with features of instability. Moreover, He et al. developed a transmission-mode mid-IR PAM system working in the wavelength range of 2.5 to 12 μm [59]. Employing its high sensitivity to optical absorption and low ultrasonic attenuation of tissue, they imaged fresh coronal slices of a mouse brain. Their study found that high-contrast lipid composition images could be obtained at 2850 cm^−1^. This method can image manually sliced and thick samples without additional processing. It streamlines the imaging process and has great potential in fast histological analysis. To realize the miniaturization and low cost of the equipment, Dasa et al. developed a cost-effective high pulse energy supercontinuum source [60]. Combined with a tunable filter, they successfully demonstrated the difference in the PA spectra of cholesterol and lipids in adipose tissue in 1650–1850 nm bands.

### 3.3. Non-Invasive Monitoring of Blood Oxygen by Photoacoustic Spectroscopy

Oxygen saturation (sO_2_) is defined as the ratio between the concentration of oxygenated hemoglobin (HbO_2_) and the concentration of total hemoglobin (HbO_2_ + HbR), which is a crucial physiological parameter [61]. The absolute oxygen saturation directly affects the human respiratory and circulatory systems [62,63], and is related to cancer, heart disease, and diabetes.

The multi-wavelength method has been widely used in the measurement of sO_2_. Choi et al. proposed wavelength-modulated differential photoacoustic spectroscopy (WM-DPAS) to estimate hemoglobin oxygenation levels [64]. This technique makes use of the difference between the molar extinction coefficients of oxyhemoglobin and deoxyhemoglobin at 680 and 808 nm, using lasers of these two wavelengths to excite the blood sample. As a result, the system noise and other background noise are effectively suppressed, and the difference between the two signals is amplified. By measuring blood oxygen saturation at different wavelengths, Wei et al. reported that the combination of 660 and 805 nm has better measurement accuracy than any other combination for blood oxygen saturation detection [65]. Kiguna et al. utilized lasers at 750 and 800 nm to measure the PA signal of blood samples of rabbits, and then calculated the oxygen saturation from the obtained PA signal by two calibration curves [66]. Simultaneously, they used the oxygen saturation obtained by blood gas analysis as a reference to evaluate the accuracy of the experimental results using regression analysis and Bland–Altman analysis, which validated the reliability of the 750 and 800 nm wavelength lasers for estimating the oxygen saturation. Many studies have also been reported, which used lasers of two different wavelengths to determine oxygen saturation.

**Figure 5:**
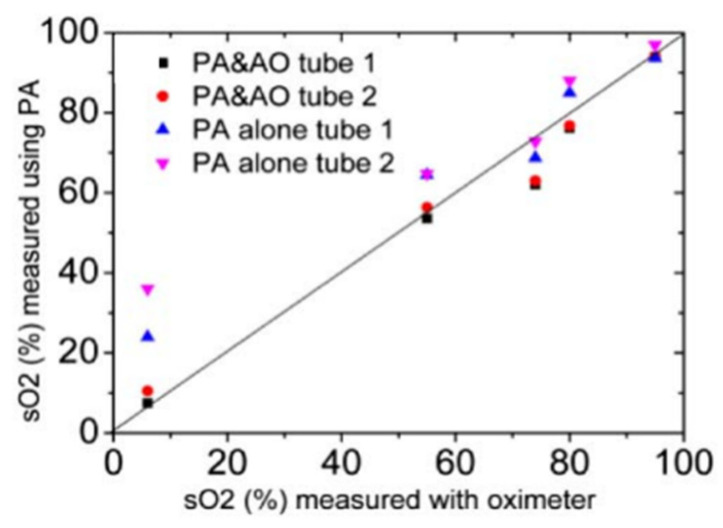
Comparison of the estimated sO_2_ using PA alone and AO-assisted fluence-compensated PA versus sO_2_ measured with an oximeter; the solid line represents the exact estimation. Reprinted from [67].

Some unconventional methods have also been reported. Hussain et al. reported a method to detect the absolute oxygen saturation of hemoglobin by combining photoacoustics (PA) and acousto-optics (AO), which utilized the AO-assisted fluence-compensated PA measurements at two wavelengths to estimate the absolute sO_2_ [67]. They used this method to measure the blood with different sO_2_ in the test tubes and compared the experimental results with the measurement results of the blood oximeter, which proves that this method can effectively estimate sO_2_. Figure 5 shows the detection of the sO_2_ value of five blood samples using PA alone and PA and AO, and are plotted against sO_2_ values measured by the oximeter. It is obvious from Figure 5 that the estimated results obtained by PA alone have a large bias from the measured results of the oximeter, whereas the values obtained by PA combined with AO are very consistent with the measured values of the oximeter.

Furthermore, Gao et al. proposed a method that combines photoacoustic and light scattering signals to monitor sO_2_ with single wavelength continuous detection [68]. This method is simple and highly integrated, showing great potential in portable monitoring. Lashkari et al. implemented a method using the phase difference between two PA signals at distinct wavelengths to estimate blood oxygenation levels [69]. Compared with the traditional PA amplitude method, the phase method is less sensitive to the absorption of laser light in the surrounding media, and therefore has lower measurement error.

### 3.4. Non-Invasive Monitoring of Cancer Cells by Photoacoustic Spectroscopy

In recent years, cancer incidence has been gradually increasing, and nearly 20 million people worldwide have cancer. The primary cancer detection methods are X-ray, CT, and MRI. Still, their defects, such as low resolution and low specificity, have troubled clinicians in diagnosing and treating cancer. PAS has advantages in safety and resolution as it is a new sensing and imaging technology, so it attracts growing research interests and is expected to complement the currently available cancer detection tools.

**Figure 6:**
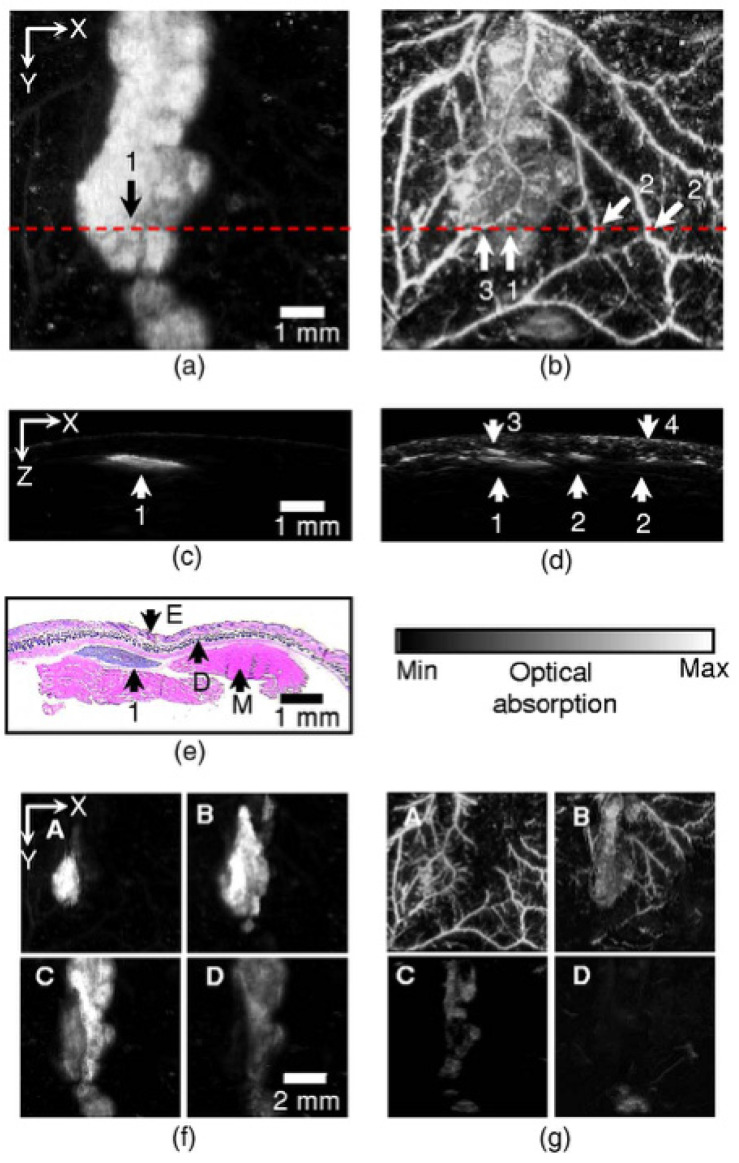
In vivo non-invasive photoacoustic images of melanoma and vascular distribution in nude mouse skin. (**a**,**b**) Enface photoacoustic images for the NIR light source (λ = 764 nm) and visible light source (λ = 584 nm), respectively: 1, melanoma; 2, vessels perpendicular to the image plane; 3, vessels horizontal to the image plane; 4, skin. (**c**,**d**) Photoacoustic B-scan images from the NIR and visible light sources, respectively, for the dot lines in (**a**,**b**). (**e**) A cross-sectional histology image (H&E staining): E, epidermis; D, dermis; M, muscle. (**f**,**g**) Depthwise enface photoacoustic images from the NIR and visible light sources, respectively; A, 0.15–0.30 mm; B, 0.30–0.45 mm; C, 0.45–0.60 mm; D, 0.60–0.75 mm from the skin surface. Reprinted from [70].

Oh et al. used a dual-wavelength reflection microscope to non-invasively image subcutaneous melanoma and its surrounding blood vessels in nude mice [70]. Because of the difference in light absorption between blood and melanin, they used two different laser wavelengths to image the blood vessels around melanin and melanoma. Only the distribution of melanoma can be obtained under near-infrared light (λ = 764 nm), since the photoacoustic signal generated by the blood vessel is lower than the system noise level. Under visible light (λ = 584 nm), the photoacoustic signal from blood vessels is 1.5–2.5 times higher than that from melanoma, so melanoma and blood vessel images can be obtained simultaneously. Figure 6 shows photoacoustic images of subcutaneous melanoma and its surrounding blood vessels in nude mice skin. Figure 6a,b shows photoacoustic images of the subcutaneous tissue of nude mice excited by a near-infrared source (λ = 764 nm) and a visible light source (λ = 584 nm), respectively. Figure 6c,d are B-scan images from the NIR and visible light sources, respectively. Figure 6e is the histological cross-sectional image (H&E staining) of skin containing melanoma in subcutaneous tissue. Figure 6f,g shows multiple maximum intensity projection images of the NIR source (λ = 764 nm) and visible light sources (λ = 584 nm) at several depths from the skin surface.

Galanzha et al. used a photoacoustic flow cytometry platform with a 1700 nm laser to non-invasively detect circulating tumor cells (CTCs) in patients with melanoma [71]. They processed the photoacoustic signal with a fast signal processing algorithm to reduce the impact of skin pigmentation and motion on the experiment. Compared with the existing CTC detection methods, their method has the ability to improve the detection limit by ~1000 times, ranging down to 1 CTC/liter of blood. Moreover, this method could detect individual CTCs at a concentration of ≥1 CTC/mL in 20 s. Their method shows the potential for early melanoma screening that deserves clinicians’ attention.

Several methods for monitoring tumor development by processing PA signals have also been reported recently. Rodrigues et al. monitored the development of breast tumors in nude mice by utilizing PAS combined with support vector machine analysis [72]. Priya et al. reported a method using wavelet principal component analysis-based logistic regression analysis to assess the tumor growth in mice [73]. Deng et al. reviewed that PA imaging combined with deep learning can play an important role in cancer diagnosis and treatment [74]. Deep learning has powerful capability in information extraction, fusion and high-speed processing, which bring new development opportunities to photoacoustic imaging.

## 4. Discussion and Conclusions

PAS combines the advantages of high optical contrast and high acoustic penetration, which is very suitable for biological tissues with non-uniform optical properties but relatively uniform acoustic properties. It can be widely used to detect blood components in tissue and organs in animals and humans, such as blood glucose detection, blood oxygen measurement, atherosclerosis imaging, cancer cell detection, etc. Another advantage of PAS is that the amplitude and phase of the photoacoustic signal can be detected directly by the target under test without pretreatment, which not only makes the operation simple but also preserves the original state of the target under test, minimizing the damage to the organism while providing more accurate results. Therefore, more and more researchers focus on applying PAS in non-invasive detection, which has brought full development to PAS technology.

However, although PAS technology has been dramatically developed, many limitations need to be improved. For clinical application, PAS technology needs to consider many problems, such as the interference of environmental factors, the specificity of detection equipment and detection objects, and the miniaturization and low cost of the equipment. At present, most studies on applying PAS to the detection of blood glucose concentration are based on glucose solution and in vitro blood samples. During in vivo detection, other substances in the blood, such as protein and lipids, will also generate signals, affecting the detection of blood glucose. In addition, considering that each tester’s skin roughness, thickness, and moisture content are different and the impact of environmental changes, such as temperature, humidity, air pressure, and the interference caused by background noise, the technology is only applicable to specific populations. Due to the lack of a general model, different samples need to be calibrated separately before testing, which brings instability to this technique. Repeatability and reproducibility are highly related to medical measurement. Therefore, how to improve repeatability and reproducibility is one of the main directions for the improvement of photoacoustic spectroscopy in the future. The problems in blood oxygen, lipid, plaque, and tumor cell detection are similar to those in blood glucose detection. Moreover, the high cost and bulkiness of the measurement system restrict the technology from being widely adopted. Therefore, these issues need to be resolved before PAS can be brought to the market. In view of the interference of complex components in blood and the human body, the subsequent research can consider combining neural networks and machine learning to denoise the extracted photoacoustic signal and improve the accuracy of the detection results. Meanwhile, future research should also focus on miniaturization and portability to develop wearable devices and substantially reduce costs. To reduce the cost and realize the miniaturization of the system, researchers in this field can cooperate with materials science researchers to develop and improve the manufacturing technology of laser and acoustic sensors. Furthermore, the measurement and imaging speed should be enhanced by exploring new scanning mechanisms and measurement schemes.

In summary, PAS has shown its advantages and potential in non-invasive human health monitoring. Although there are limitations, such as insufficient detection accuracy and sensitivity, and a high system cost, researchers are gradually finding appropriate solutions and exploring its great potential for real-world applications.

## Figures and Tables

**Table 1:** Comparison of different PAS methods.

Technology		Wavelength	Range	Target	Measurement Error
Continuous wavelength	[42]	8200–10,000 nm	50–300 mg/dL	Skin/in vivo	7–15 mg/dL (RMSE)
	[43]	9130–9900 nm	>100 mg/dL	ISF/in vitro	-
	[44]	9130–9900 nm	>57 mg/dL	Glucose solution and skin/in vitro and in vivo	-
	[45]	8000–10,000 nm	75–160 mg/dL	Skin/in vivo	16% (outside of the clinical region based on the Clarke grid [51])
Dual wavelength	[21]	1382 and 1610 nm	50–300 mg/dL	Skin/in vivo	19–48 mg/dL (standard error), <10% (outside of the clinical region based on the Clarke grid)
Single wavelength	[48]	1600 nm	>30 mg/dL	Human blood serum/in vitro	9.84 mg/dL (RMSE), 14.9 mg/dL (standard deviation)
